# The Treatment of Whooping-Cough with Atropia Used Hypodermically and Carbolic Acid Inhalations

**Published:** 1880-09

**Authors:** Wm. Lee


THE TREATMENT OF WHOOPING-COUGH WITH ATROPIA USED HYPODERMICALLY AND CARBOLIC ACID INHALATIONS, BY WM. LEE, M. D.

In August, 1879, having under my care a number of cases of whooping-cough, in some of which the paroxysms were unusually severe, I determined to try this plan of treatment, which, in part I had shortly before seen highly recommended in *The Lancet* and in *The London Medical Record,*—the difference being, that I used the atropia hypodermically, instead of giving it by the mouth, as recommended in *The Lancet.* I did so because of my great faith in hypodermic medication; because the dose of atropia, which is unvarying in its strength, is easily regulated; and because the result of all investigation in regard to its action shows not only that cutaneous sensibility is rapidly lowered by it, but that at the same time an anæsthetic effect is produced upon the afferent branches of nerves which originate spasms.

Each minim of the solution used contained $\frac{1}{120}$ of a grain of atropia. I injected one minim or more, according to the patient's age, with 10 minims of water, always using it as early in the morning as possible, and repeating it at night if occasion required. The carbolic acid solution, of the strength of five per cent., made with the very best crystals, was used as follows: five strips of Canton flannel, three inches wide and five inches long, were saturated with this solution, and hung around the patient's bed and about the room at bedtime, and they were moistened with the solution once again during the night.

The result of the treatment in these cases justifies the belief, I think, that with it we may expect a steady diminution in the number and the duration of the paroxysms, a change in the character of the whoop, and a cure of the disease in a much shorter time than has been accomplished by any other means.

Out of several cases successfully treated in this way, I report two in detail:

Case I.—Mary S——, three years old, first seen August 1st, had well-developed whooping-cough. The mother stated that she had whooped fifteen times daily for the previous three days. She had particularly noticed each one because of their being so severe. One minim of the atropia solution was injected, and the carbolic acid was used at bedtime. August 2d. Since the last note she has had ten paroxysms, those at night having been less severe. 3d.—There have been six paroxysms since the last visit—one, at night, having been very severe. 4th.—Six paroxysms have occurred, two of them very severe. The nurse had neglected the inhalations. 5th.—There have been four paroxysms, of short duration. 6th.—Three paroxysms, of very slight severity, have occurred. 7th.—But one paroxysm has occurred; very severe, however. 8th—There have been no more paroxysms. She is very thirsty. The treatment, which had consisted of the daily repetition of that mentioned in the first note, was now suspended. 9th.—There have been two paroxysms. Treatment renewed. 10th.—She has had no paroxysms, and has coughed but little. At the end of three days more she had entirely recovered.

Case II.—John K——, five years old, was first seen August 14th, at night. The mother said that he had been sick for some time, and that, as well as she could remember, he had had twelve paroxysms daily for two days previously. The atropia and carbolic acid treatment was begun at once. 15th.—Ten paroxysms have occurred, 16th.—There have been nine paroxysms—so severe that a second injection had to be given. 17th.—He has had six paroxysms, much less severe. 18th—There have been four paroxysms—one, at night, having been very severe. The use of the carbolic acid had been neglected. 19.—Three paroxysms have occurred, shorter and milder. 20th.—One paroxysm. 21st.—He has had four paroxysms. He seems to have taken cold, and has high fever. The breathing is very short, but no pulmonary complication is discovered. The injection was omitted, but the use of carbolic acid was continued, and he was ordered small doses of spiritus minderei. 22d.—There is no longer any fever. The paroxysms have been six in number, but they have not been so severe as before. The injections were resumed, and the spiritus minderei was discontinued. 23d—Three very mild paroxysms have occurred. 24th.—One paroxysm. 25th.—No further paroxysms have occurred. The treatment was now stopped, and in four days he was well.—*New York Medical Journal.*

